# Polarity Establishment and Maintenance in Ascidian Notochord

**DOI:** 10.3389/fcell.2020.597446

**Published:** 2020-10-30

**Authors:** Hongzhe Peng, Runyu Qiao, Bo Dong

**Affiliations:** ^1^Sars-Fang Centre, MoE Key Laboratory of Marine Genetics and Breeding, College of Marine Life Sciences, Ocean University of China, Qingdao, China; ^2^Laboratory for Marine Biology and Biotechnology, Qingdao National Laboratory for Marine Science and Technology, Qingdao, China; ^3^Institute of Evolution and Marine Biodiversity, Ocean University of China, Qingdao, China

**Keywords:** notochord, planar cell polarity, apical-basal polarity, initiation of AB polarity, lumen formation

## Abstract

Cell and tissue polarity due to the extracellular signaling and intracellular gene cascades, in turn, signals the directed cell behaviors and asymmetric tissue architectures that play a crucial role in organogenesis and embryogenesis. The notochord is a characteristic midline organ in chordate embryos that supports the body structure and produces positioning signaling. This review summarizes cellular and tissue-level polarities during notochord development in ascidians. At the early stage, planar cell polarity (PCP) is initialized, which drives cell convergence extension and migration to form a rod-like structure. Subsequently, the notochord undergoes a mesenchymal-epithelial transition, becoming an unusual epithelium in which cells have two opposing apical domains facing the extracellular lumen deposited between adjacent notochord cells controlled by apical-basal (AB) polarity. Cytoskeleton distribution is one of the main downstream events of cell polarity. Some cytoskeleton polarity patterns are a consequence of PCP: however, an additional polarized cytoskeleton, together with Rho signaling, might serve as a guide for correct AB polarity initiation in the notochord. In addition, the notochord’s mechanical properties are associated with polarity establishment and transformation, which bridge signaling regulation and tissue mechanical properties that enable the coordinated organogenesis during embryo development.

## Introduction

Cell proliferation, migration, and deformation; extracellular matrix (ECM) secretion; and cell–cell junction formation and remodeling are basic processes in tissue and organ architecture establishment during embryogenesis. However, these processes are not always isotropic, and anisotropic processes lead to spatial differences in cell shape and structure, directed cell behaviors (e.g., migration, mitosis, adhesion, secretion, and signaling transition), and asymmetric subcellular structures (e.g., organelle localization and cytoskeleton distribution) all of which generate what is called “polarity” (Dhonukshe, 2013; Wolpert, 2013). Polarity widely exists during the entire lifespan and at all levels of an organism. At the tissue level, polarity appears as asymmetric differentiation forming distinct cell types and/or the mechanically anisotropic tissues. For the whole embryo, polarity manifests as the formation of orientated body axis and the asymmetric location of organs.

Polarity is regulated by signaling pathways that respond to chemical stimulations such as morphogens (Turing, 1952; Pickett et al., 2019) or physical stimulation like light (Brownlee and Bouget, 1998) from the extracellular space, where oriented signals are sent, except a small part of spontaneous random cell polarization (Wedlich-Soldner and Li, 2003). Extracellular signals transmit through paracrine pathways mediated by morphogen concentration gradient signaling, such as Wnt signaling (Ukita et al., 2009), Notch signaling (Hermann et al., 2000; Kato, 2011), Hedgehog signaling (Choi and Harfe, 2011; Hu et al., 2017), and transforming growth factor beta signaling (bone morphogenetic protein, activin) (Ninomiya, 2004; Eom et al., 2011). In addition, cells also receive orientation signals through contacts, such as cell–cell adhesion (Nance, 2014) or cell-ECM network like basement membrane adhesion (Marsden and DeSimone, 2003). These extracellular signals provide directional information, which induces asymmetric distribution of protein complexes or subcellular organelles, establishing intracellular polarity (Pickett et al., 2019). There are two main intracellular polarity signal systems, apical-basal (AB) polarity mediated by the Par/atypical protein kinase C (aPKC) complex (Assémat et al., 2008), and planar cell polarity (PCP), mediated by Frizzled (Fz, also called Fzd in vertebrate)- Flamingo (Fmi, also known as starry night in Drosophila and Celsr in vertebrate)- Disheveled (Dsh, also called Dvl in vertebrate)- Diego (Dgo, also called Diversin or Inversin in vertebrate), and Van Gogh (Vang, also known as strabismus in Drosophila and Vangl in vertebrate)- Flamingo (Fmi)- Prickle (Pk, also known as prickle-spiny legs in Drosophila) complexes (Adler, 2012; Yang and Mlodzik, 2015). Both intracellular polarity signal systems coexist in cells and sometimes cooperate too (Djiane et al., 2005; Wu and Mlodzik, 2009), contributing to the formation and maintenance of multidimensional polarity.

Polarity establishment is necessary for morphogenesis, by which three-dimensional (3D) complex architectures emerge from symmetric, simple structures. Tissue and organ specialization and cell fate depend on the direction and position information. For example, anterior-posterior (AP) axis formation regulates somite differentiation (Bénazéraf and Pourquié, 2013), while left-right (LR) axis formation regulates the asymmetry location and differentiation of the brain, heart, and gut (Bisgrove et al., 2000; Hashimoto and Hamada, 2010; Grimes, 2019). The body plan symmetry of animals is diversified including spherical symmetry, radial symmetry, biradial symmetry, and bilateral symmetry (Holló, 2015). In bilaterally symmetric animals, some inner organs, such as the heart and gut, in the symmetric body plan show LR asymmetry (Raya and Belmonte, 2006).

Of multi-leveled polarities, tissue polarity, formed from cell polarity (Mlodzik, 2002) and manifesting as tissue mechanical property and tissue movement, epitomizes the polarity phenomena and plays an important role in developmental processes.

## The Notochord

The notochord is a characteristic rod-like midline organ in chordate embryos that supports the body structure and produces signaling (Stemple, 2005; Corallo et al., 2015). Notochord evolution has three stages: muscle-like axochord (Lauri et al., 2014; Brunet et al., 2015), rigid cell cord, and osseous vertebral column. A notochord-like muscle structure has been reported in annelids (Lauri et al., 2014) indicating that the notochord originates from muscle (Lauri et al., 2014; Brunet et al., 2015). In hemichordatas, the lowest chordates, the stomochord, a notochord primordium, appears (Balser and Ruppert, 1990). In amphioxus, a cephalochordate, the notochord is present throughout the body during the entire lifespan as the body’s main support structure (Feng et al., 2016). In urochordates, such as ascidians, the notochord only exists in the tail part in swimming larvae and disappears after metamorphosis (Cloney, 1982; Matsunobu and Sasakura, 2015). In cyclostomes, cartilaginous tissue appears around the notochord (Ota and Kuratani, 2007). In higher chordates, such as fish and mouse, the notochord exists centrally in the embryonic and larval body, and it is replaced by the spine in adults (Wopat et al., 2018; Bagwell et al., 2020). The notochord is converted into the nucleus pulposus in fully formed intervertebral discs, which protect the vertebrae from rubbing against each other (McCann et al., 2011).

## Polarities in *Ciona* Notochord

**FIGURE 1:**
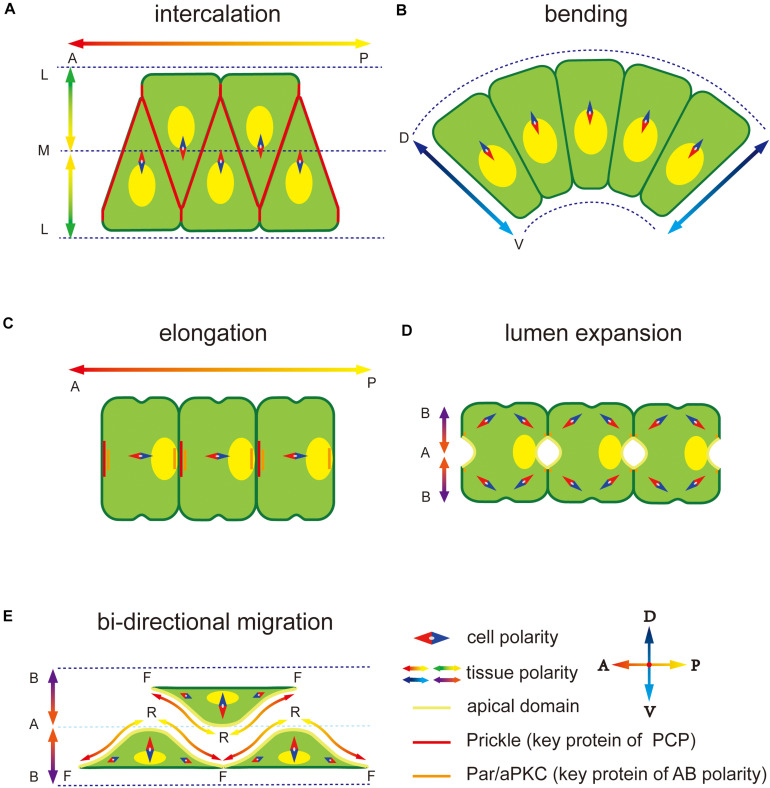
Polarity at different notochord development stages in *Ciona*. **(A)** Notochord cells form a mediolateral (ML) polarity to drive the migration to the midline during convergence and extension. The notochord elongates parallel to the anterior-posterior (AP) axis regulated by embryonic AP polarity signaling. The key PCP component protein Prickle is located at the notochord cell–cell contact domain. **(B)** Notochord cells form a dorsal-ventral (DV) polarity to abend tissue toward the ventral side. **(C)** After cell intercalation, the PCP direction changes from ML to 1D AP axis. The Prickle relocates at the anterior edge of each notochord cell. **(D)** Apical-basal (AB) polarity is built to induces extracellular lumen formation. Two apical domains appear in one notochord cell. **(E)** Notochord cells migrate bidirectionally to induce the lumen connection. Adjacent notochord cells flatten opposite to each other along the notochord sheath.

Ascidian is a basic group in chordate, with the notochord at larval stage. It is becoming an ideal model in marine invertebrates’ morphogenesis (Lv et al., 2019). During notochord development in *Ciona*, diverse polarity patterns are built and play an important role in asymmetric structure formation and directional movement of notochord cells. First, notochord cells intercalate to form a rod-like structure along the midline of the body through convergence extension (CE) process regulated by a medial-lateral (ML)-oriented PCP (Figure 1A; Munro and Odell, 2002). During CE, ventrally polarized cytoskeleton contractility induces the notochord and the tail to bend ventrally and elongate posteriorly within the chorion (Figure 1B). Next, mesenchymal-epithelial transition (MET) occurs, in which notochord cells become epithelial-like with AB polarity (Figures 1C,D). The PCP direction changes from ML to the one-dimensional (1D) AP axis (Kourakis et al., 2014). During the period, notochord cells secret the ECM into the basal, building a notochord sheath, and also into the apical surfaces, forming the extracellular lumen for cavitation (Figure 1E), which provides sufficient stiffness to support the body (Keller, 2006; Yasuoka, 2020). Polarity plays an essential role in all these processes. This review focuses on MET of notochord cells and polarity establishment and maintenance during this process.

## Establishment of AB Polarity in *Ciona* Notochord

A rod-like notochord appears after cell intercalation, in which PCP signaling sends polar signals parallel to the AP axis (Kourakis et al., 2014). The notochord elongates and undergoes MET, establishing AB polarity (Dong et al., 2009; Denker et al., 2013). The MET includes two main steps: (1) apical domains initially appear at the center of two opposite lateral domains of each notochord cell; (2) extracellular lumen is deposited outside of apical domains and expanded continuedly (Denker and Jiang, 2012). The Par3-Par6-aPKC polarity complex is essential for AB polarity formation in epithelial cells (Assémat et al., 2008; Dhonukshe, 2013; Wen and Zhang, 2018). In *Ciona* notochord, Par3, Par6, and aPKC co-localize at the AP polar edges of notochord cells. Inhibition of Par3 function causes loss of the polarized localization of Par6 and aPKC, and the lumen cannot form, suggesting that Par3 might be the signaling upstream to guide Par/aPKC and AB polarity formation. It has been known that knockdown of *Par3* caused mislocalization of Par6 and aPKC, and blocked the AB polarity formation. However, mislocalization of Par6 using an aPKC binding dominant negative (PAR-6 ΔaPKC-BD) (Suzuki et al., 2001) did not affect the localization of Par3. While, overexpression of myristoylated aPKC (myr-aPKC), which leads to the mislocalization of aPKC, caused the mislocalization of Par6. However, the localization of Par3 was unaffected (Denker et al., 2013). These results indicate that Par3 is upstream of aPKC and Par6, while the localization of Par6 depends on aPKC in *Ciona* notochord. The upstream signaling that induces the Par3 polar localization is still unknown. Some studies in *Caenorhabditis elegans* and *Drosophila* suggest that the localization of Par/aPKC complex is regulated by centrosome (Feldman and Priess, 2012). Engulfment and cell mobility (ELMO) family proteins might be involved in the regulatory process (Schmidt et al., 2018). With the development, ECM components are secreted out, forming the extracellular lumen at the anterior and posterior edges of notochord cells, and the notochord cell surface is separated into apical (facing the lumen), lateral (contact between neighboring cells), and basal membrane domains. After lumen formation, the Par3-Par6-aPKC polar complex localizes at the boundary between apical and lateral membranes to form a ring-like localization (Denker et al., 2013; Smith, 2018). Initially, each cell forms two apical domains in the notochord in *Ciona*, which then eventually merge into one (Figures 1D,E; Dong et al., 2009). During this process, notochord cells are regulated by polar signaling from both PCP and AB polarity that induces many proteins, such as cytoskeleton and cell junction proteins, toward polar localization. A contractile ring appears at the anterior side of each notochord cell and then moves to the equator to provide the force required for cell elongation (Dong et al., 2011). Zonula Occludens 1 (ZO-1), as part of the tight junction, co-localizes with the Par3-Par6-aPKC polar complex and they work together to occlude the lumen cavity (Denker et al., 2013). Solute Carrier 26aα (SLC26aα), a transmembrane transport protein locates at the apical membrane to control the osmotic pressure in the lumen cavity (Deng et al., 2013).

The relationship between PCP and AB polarities, that is, whether and how they cross-talk, is still unclear. There exist three possible ways of communication: (1) Key PCP and AB polar proteins might interact directly. This type of cross-talk between PCP and AB polarities has been identified in the *Drosophila* eye, in which aPKC inhibits PCP activity by Fz1 phosphorylation (Djiane et al., 2005; Wu and Mlodzik, 2009). In *Ciona* notochord cell, Prickle and Par/aPKC complex are overlapped at the presumptive anterior apical domain (Jiang et al., 2005; Denker et al., 2013), offering the possibility for their direct interaction. (2) Notochord cells receive signals from neighboring cells. In intercalation abnormal mutant *Ciona* embryos, a notochord cell is usually in contact with more than two adjacent cells, and apical domains can be observed at each cell–cell contact (Denker et al., 2013), confirming that the AB polarity of notochord cells is affected by neighboring cells. When cell ablation removes the posterior neighboring cell, the notochord cell loses the posterior location of the nuclei. In addition, nucleus in the 40th notochord cell, which is the last cell in notochord tissue, locates in the anterior part of the cell (Kourakis et al., 2014). Both the observed facts indicate that maintenance of PCP signaling is posterior cell dependent. (3) ECM components mediate the interaction between PCP and AB polarities. Disruption of PCP signaling causes the mislocation of ECM component at the cell–cell interface (Veeman et al., 2007), suggesting that localization of basal ECM is PCP dependent. In addition, in zebrafish and *Xenopus*, disruption of the function of notochord sheath protein components, such as laminin or collagen, leads to abnormal notochord and vacuole morphogenesis (Parsons et al., 2002; Pagnon-Minot et al., 2008; Buisson et al., 2014; Manninen, 2015).

## Polarized ECM Secretion in the Notochord

Notochord tissue in different species has a few common features, such as the notochord sheath (closed tube component of the ECM) and the lumen cavity formed by distinct ways. In *Ciona*, notochord cells form an extracellular lumen through MET, surrounded by a single layer of notochord cells and an ECM notochord sheath (Dong et al., 2009). In zebrafish, some notochord cells form a layer of outer notochord sheath cells along the notochord sheath while other cells move to an inner cell core, and vacuoles are formed within each cell (Ellis et al., 2013a,b; Corallo et al., 2015). In amniotes, such as chicken and mice, vacuolated notochord cells are surrounded by an acellular sheath (Choi et al., 2008; Ward et al., 2018).

Notochord sheath formation is oriented by secretion of the polarized basal surface ECM. In *Xenopus*, the chordamesoblast forms radial polarization induced by surrounding tissues and then secretes fibronectin at the basement surface, forming a fibronectin layer between the mesoderm and surrounding tissues. Disruption of the Pk and/or Stbm/Fz function leads to failure of basal ECM secretion (Goto et al., 2005), indicating that the Wnt/PCP signaling pathway is essential for basal ECM secretion. Similarly, failure of laminin (polarized ECM component) secretion is found in *Pk* mutant *Ciona* embryos (Veeman et al., 2007; Veeman and McDonald, 2016), indicating that a conserved regulatory mechanism underlies basal ECM secretion. In addition to the Wnt/PCP signaling pathway, other signaling pathways, such as hedgehog, might also be involved in notochord sheath formation (Choi and Harfe, 2011).

In addition to basal ECM secretion, apical ECM secretion is also found sometimes (Mizotani et al., 2018; Bhattachan et al., 2020), which forms extracellular pocket lumens. During this process, notochord polarity moves from PCP to AB polarity (Dong et al., 2009). Both PCP an AB polarities and basal vesical trafficking are required for apical ECM secretion (Mizotani et al., 2018).

## Cytoskeleton Polarities in *Ciona* Notochord Morphogenesis

The cytoskeleton, including the microtubule, actin filaments, and intermediate filaments, is a group of fibriform intracellular proteins that form a skeleton network to maintain the cell shape and movement (Pegoraro et al., 2017). Cytoskeleton polarity, such as asymmetry distribution; oriented arrangement; and polar crosslinking, cooperation, disassociation, and rearrangement, plays an important role in cell asymmetry structures and behaviors. A polarized cytoskeleton is the main cause of an unbalanced bioforce. In contrast, a cytoskeleton is highly dynamic. It can be sensitively regulated and under the control of cell polarity (Raman et al., 2018). Therefore, cytoskeleton polarity is an important pathway to externalize intracellular polar signaling.

**FIGURE 2:**
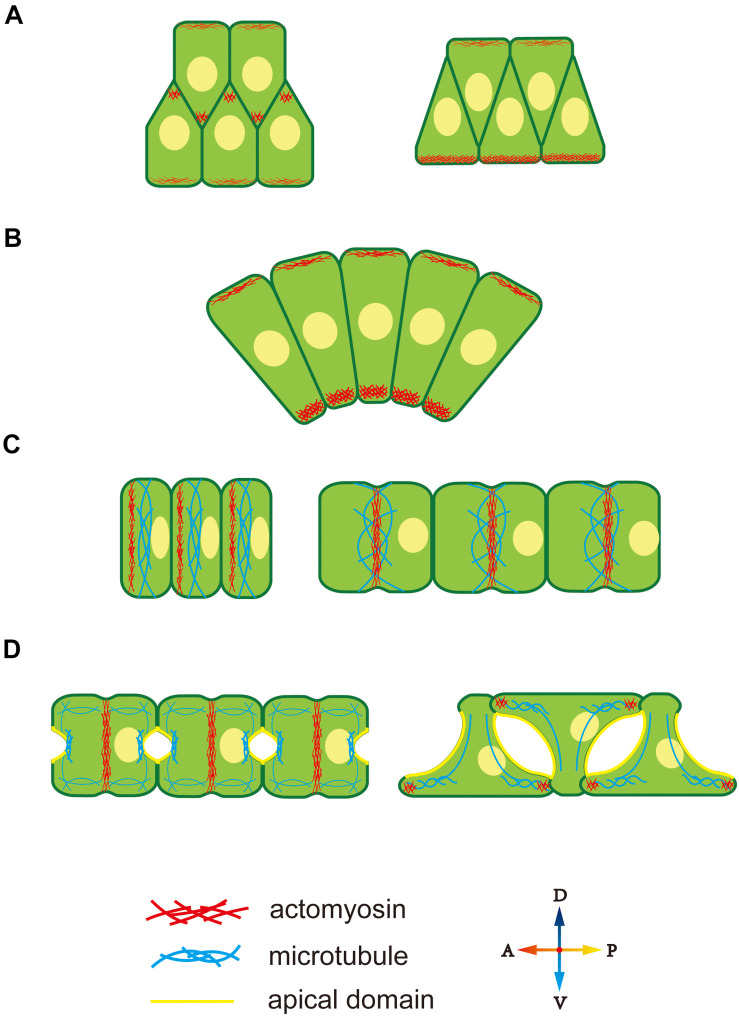
Cytoskeleton polarity during notochord development in *Ciona*. **(A)** F-actin accumulates at the lamellipodium tip, providing migrating forces for cell intercalation. **(B)** Ventrally accumulated actomyosin contractility provides unbalanced force to drive notochord bending. **(C)** An actomyosin contractile ring forms at the anterior edge and moves to the equator of notochord cells. Actomyosin ring contraction elongates notochord cells. Microtubules are perpendicular to the AP axis within notochord cells. **(D)** During lumen expansion, microtubules accumulate at the apical domain, and along with bidirectional migration, they rotate 90° and form oriented bundles toward the leading edges of tractive lamellipodia-like protrusions. An actomyosin contractile ring also exists during lumen expansion and then disappears. At bidirectional migration, F-actin moves to the tip of lamellipodia-like protrusions.

Cytoskeleton polarity plays an important role in diverse notochord morphogenesis, including the ascidian notochord. At the early stage of notochord development in *Ciona* notochord, cytoskeleton distribution is regulated by the PCP signaling pathway (Sasakura and Makabe, 2001; Niwano et al., 2009), which induces accumulation of midline-oriented F-actin at the membrane cortex, forming cell protrusions (Figure 2A) (Munro and Odell, 2002). These protrusions generate a mechanical force to drive cell extension and migration across the surfaces of neighbor notochord cells toward the midline. Disruption of the function of PCP components, causes notochord cells to move in random directions instead of toward the midline (Jiang et al., 2005).

During CE, the notochord bends ventrally to adapt to the limited space inside the chorion. During this process, both actin and myosin II present an obviously ventral-polarized distribution in notochord cells. The dorsal-ventral (DV) polarity of the cytoskeleton in the notochord is observed during cell intercalation (Figure 2A). At this stage, notochord cells are arranged in two rows, while, actomyosin accumulates at the ventral cortex of the ventral row, that is, the ventral boundary of notochord tissue (Figure 2A, right panel). The polarized actomyosin generates a mechanical force that, along with the faster dorsal epidermis proliferation, drives notochord bending, therefore, embryonic tail bends. The most obvious actomyosin polarity appears at the anterior part of the notochord, where the bending angle is largest (Figure 2B). The cytoskeleton DV polarity can only be found in the ventral row during cell intercalation. However, when notochord cells in the dorsal row migrate across the ventral row, they immediately get DV polarity, indicating that the dorsal and ventral rows of notochord cell populations build DV polarity at different time and that establishment of cytoskeleton DV polarity in notochord cells depends on whether the cells are in contact with the ventral boundary of notochord tissue. The signaling pathway that controls DV-polarized cytoskeleton is unclear. The ECM protein laminin localizes dorsally (Veeman et al., 2007), while the apical cell polarity molecule aPKC localized ventrally (Oda-Ishii et al., 2010), which might provide a polarizing signal for polarized actomyosin enrichment. The aPKC has been demonstrated to phosphorylate Lgl to inhibit its interaction with myosin II (Betschinger et al., 2003), providing a cue for further study on the mechanism how D-V polarity influences cell behavior.

After CE, F-actin, and non-muscle myosin present as an actomyosin contractile ring perpendicular to the AP axis to drive notochord cell elongation (Dong et al., 2011; Sehring et al., 2014; Lu et al., 2019). The actomyosin contractile ring first appears at the anterior edge of the notochord and then migrates toward the equator (Figure 2C). Positioning of the actomyosin contractile ring is regulated by a “tug-of-war” mechanism between PCP signaling and actomyosin contractility. Inhibition of contractility causes the return of contractile ring to the anterior side, while disruption of both PCP and contractility causes random positioning of contractile ring, indicating that the PCP determines the initial ring position and contractility drives the ring to move to the equator (Sehring et al., 2015). More actomyosin accumulates at the anterior edge compared to the posterior membrane (Newman-Smith et al., 2015). The anterior actomyosin cytoskeleton and core PCP component mutually co-localize at the anterior membrane domain. They interact and maintain the stable 1D PCP along the AP axis. The cytoskeleton is controlled by cell polarity; conversely, cytoskeleton polarity regulates the localization of key polarity-signaling molecules.

After notochord cell elongation, an extracellular lumen forms and then expands between adjacent notochord cells (Dong et al., 2009). This process begins with PCP-AB polarity transition (Denker et al., 2013). The mechanism underlying polarity transition is unclear, but cytoskeleton polarity is likely involved. AB polarity formation in *Drosophila* depends on the F-actin cytoskeleton (Schmidt et al., 2018; Sokac and Wieschaus, 2008a,b). In notochord cells in *Ciona*, a polarized microtubule network is seen toward the apical membrane, which might signal AB polarity formation (Figure 2D). Cortical actin and ERM are found to be essential for lumen formation (Dong et al., 2011). This cytoskeleton network might function as a vesicle-trafficking track to secret the ECM into the extracellular lumen (Mizotani et al., 2018; Bhattachan et al., 2020).

When the lumen expands to a specific volume, notochord cells begin to migrate bidirectionally and flatten (Figure 2D). Lamellipodia-like protrusions, whose formation depends on the F-actin network, provide the mechanical force for migration (Dong et al., 2011). Disruption of F-actin polymerization causes the protrusions to disappear and notochord cells to lose migration ability. At the stage of protrusion formation, the microtubule cytoskeleton rotates 90° and forms oriented bundles toward the leading edges of tractive lamellipodia (Dong et al., 2011). Inhibiting microtubule assembling leads to abnormal the location and number of cell protrusions, indicating that polarized microtubule provides directional information for notochord cell migration.

## Polarity Signaling Regulates Mechanical Properties of the Notochord Tissue

Development is a coordinated multi-tissue-reshaping and movement process. It is also a process of biomechanical force generation, conduction, and release. During development, different tissues have distinct mechanical properties, leading to different reshaping and migratory patterns. A tissue, as a type of specific biomaterial, has autokinetic movement ability, which is a characteristic of life. Therefore a tissue’s mechanical properties include not only a shape-changing ability under force, such as bending, stretching, compressing, and twisting, which are described by materials science, but also a regulatory ability to magnify, locate, and orientate bioforces (Mammoto and Ingber, 2010). The tissue’s mechanical properties are determined by diverse bioforces generated by subcellular or supracellular structures, such as the cytoskeleton, nuclei, ECM, and cell junctions (Ingber, 2006). Polarity, as dynamic signaling, drives a change in a tissue’s mechanical properties by regulating these subcellular or supracellular structures to match embryo developmental processes. Therefore, polarity can serve as a bridge connecting signaling regulation and tissue mechanical properties and coordinate organogenesis during embryo development.

The notochord, as a center pillar-like structure is the main support organ of chordate embryo development (Corallo et al., 2015). In addition, the notochord is a typical polarity-induced organ, and many polarity regulatory pathways are involved in notochord formation. Therefore, the notochord is an important model to study how polarity regulates a tissue’s mechanical properties.

**FIGURE 3:**
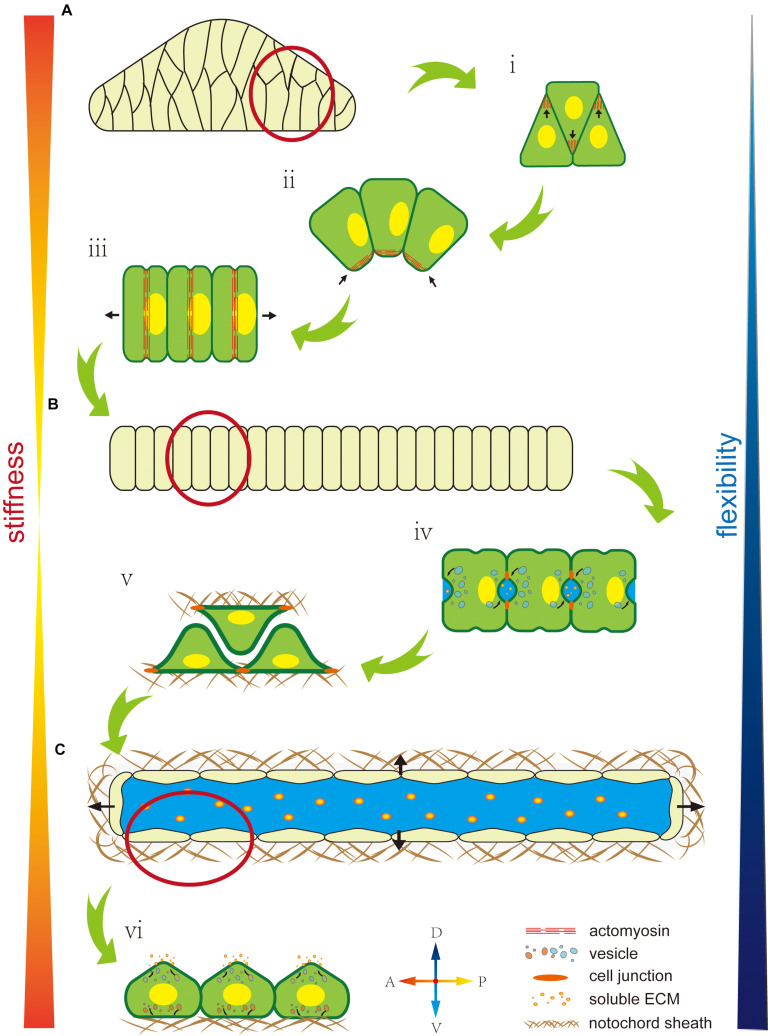
Mechanical properties of the notochord are regulated by cell polarity. **(A)** The early notochord is a short, thick, stiff, rod-like structure made up of a multiline of cells. **(i)** Polarized F-actin drives cells to migrate and intercalate to form a single line. **(ii)** Ventrally polarized actomyosin drives notochord bending toward the ventral side. **(iii)** An equator-localized contractile ring drives notochord cell elongation. **(B)** The notochord forms as a long, thin, single cell-line cord. Its stiffness is low but flexibility is high, which facilitates rapid embryonic development in the narrow space inside the chorion. **(iv)** AB polarity is built during extracellular lumen formation. **(v)** Notochord cells migrate bidirectionally along the notochord sheath. **(vi)** Notochord cells form an endothelial-like single layer. **(C)** A single lumen-filled notochord tube increases tissue stiffness and flexibility, which enables the swimming of *Ciona* larva in seawater.

At the intercellular level, polarity regulates not match with the properties cell’s mechanical properties by affecting the cytoskeleton, nuclei position, or oriented vesicle transport. The cytoskeleton, as an intracellular support structure, is the main cause of an unbalanced bioforce. A polarity signal, such as a PCP signal, regulates the Rho signal pathway to affect cytoskeleton distribution (Nishimura et al., 2012), while cytoskeleton localization and orientation creates cells’ mechanical properties (Hefele et al., 2004; Shin et al., 2007). As described earlier, F-actin accumulates at the tip of the lamellipodium to force cell migration toward the midline during cell intercalation (Figure 3Ai). In notochord cells in *Ciona*, the ventrally accumulated actomyosin contracts to generate a force that bends the notochord (Figure 3Aii). Actomyosin contractile rings localize at the middle of the cells to drive cell elongation (Figure 3Aiii). Therefore, cytoskeleton localization affects cells’ mechanical properties, while polarized oriented microtubule bundles help lumen expansion and protrusion formation, indicating how cytoskeleton orientation changes cells’ mechanical properties (Dong et al., 2011).

The asymmetric position of nuclei also regulates cells’ mechanical properties (Calero-Cuenca et al., 2018). During notochord cell elongation in *Ciona* embryos, PCP signaling drives the posterior location of the nuclei in notochord cells (Figure 3Aiii) (Jiang et al., 2005; Kourakis et al., 2014). However, the underlying mechanism is still unclear and might be related to the notochords’ mechanical properties. In amoeboid migration of immune or lobopodial cells, the nucleus’ position affects mechanical properties, such as strength and migration ability (Petrie et al., 2014; Salvermoser et al., 2018; Renkawitz et al., 2019).

In addition, polarity controls polarized membrane vesicle trafficking, which in turn affects cells’ mechanical properties. During notochord lumen expansion in *Ciona*, vesicles are transported toward the apical domain to supply the apical membrane and release the membrane tension from continuous lumen expansion (Figure 3Biv) (Mizotani et al., 2018; Bhattachan et al., 2020).

At the supracellular level, polarity can regulate oriented ECM secretion, which directly affects cells’ mechanical properties by forming a crosslinked network to provide enough strength and elasticity or indirectly regulates by contributing to lumen formation (Loganathan et al., 2020). In the notochord in *Ciona*, basal ECM secretion forms a basal lamina-like notochord sheath (Figure 3Bv) (Wei et al., 2017). In addition, some of the ECM has high hydrophilicity, significantly increasing osmotic pressure (Lu et al., 2006). Polarity signaling controls the fixed-point release of the ECM to control osmotic pressure variation in specific areas to influence extracellular liquid flow (Figure 3Cvi). There is a lot of ECM in the notochord lumen in *Ciona*, which is secreted by the apical membrane to increase osmotic pressure. Apical lumen expansion together with the basal notochord sheath, forms a fiber-wound cylinder hydrostatic skeleton. Since it is difficult to compress the liquid limen, this structure impacts a higher strength to the notochord than the limit of ECM’s and notochord cells’ mechanical properties (Adams et al., 1990; Stemple, 2005; Yasuoka, 2020). In addition, the flexibility of this fiber-wound cylinder structure is higher compared to cell reshaping and ECM remodeling. Therefore, it facilitates dynamic regulation of the notochord to adapt to faster embryo development. In *Ciona*, the notochord forms a connected single lumen. In contract, in zebrafish, the notochord forms multiple, discontinuous intracellular vacuoles (Ellis et al., 2013a,b; Corallo et al., 2015). The high connectivity of the lumen might be beneficial by dispersing the strength through lumen matrix flow, which impacts to the notochord greater impact resistance. However, this structure cannot control the shape locally. The evolutionary trend from a single lumen to multiple disconnected vacuoles might compromise the notochord stiffness but impart tissue flexibility, which makes the notochord adapt to the more complex developmental processes in vertebrates.

## Summary and Perspective

*Ciona* notochord is composing of 40 cells with diverse polarities phenomena during development, being an ideal simple model for polarity study. Besides, the *Ciona* genome is single-copy, which benefits to elucidate the molecular mechanism for polarity establishment and maintenance. Because of the specifically evolutionary position in animal phylogenetic taxa, the knowledge achieved from *Ciona* notochord polarity can be used for reference toward both invertebrate and vertebrate.

Although the *Ciona* notochord model is simple for polarity study, there still remains several challenge questions need to be answered in the future. For example, what is the signaling contributing to the establishment of the initial AB polarity? Who mediates the interaction and transition between PCP and AB polarity? How do polarities coordinate the concerted and coherent development in multiple tissues?

Par/aPKC complex contributes to the establishment of AB polarity in notochord cell. However, the signaling for the establishment of initial AB polarity is still unknown. It has been indicated that Rho signaling mediated centrosome and microtubule play an important role in the initial localization of the key component proteins of AB polarity. Further study to reveal the controlling signals for the localization of Par/aPKC complex in *Ciona* notochord cells will provide cues to understand how AB polarity is initially established in a cell.

The crosstalk between PCP and AB polarity is also worth further exploring to learn about how the different polarity systems are coordinated. Several ways including the interaction between PCP and AB polarity components, cell–cell contact, and cell-basement membrane contact are candidate pathways to mediate such crosstalk between PCP and AB polarity. However, the molecular mechanisms underlying these possibilities are unknown. Investigation in a structurally simple and single gene copy notochord system will benefit to answer the question.

Polarity plays an important role in coordination of multiple tissue development by controlling tissue mechanical property. For example, when flat cell sheets bundle to form a biological tube, polarity regulates an asymmetry cell wedge or drives cell directional intercalate, leading to the bending movement through the mechanical property change in different parts of the cell flat sheet (Nielsen et al., 2020). Compared with the anatomically complex of vertebrate, *Ciona* embryonic tail is structurally simple, providing an excellent model to understand how polarity signaling coordinates the multiple tissue development.

## Author Contributions

BD and HP conceived and wrote the manuscript. HP prepared the figures. RQ provided critical editing and input. All authors contributed to the article and approved the submitted version.

## Conflict of Interest

The authors declare that the research was conducted in the absence of any commercial or financial relationships that could be construed as a potential conflict of interest.
